# Lycopene: A Potent Antioxidant for the Amelioration of Type II Diabetes Mellitus

**DOI:** 10.3390/molecules27072335

**Published:** 2022-04-04

**Authors:** Hui Eng Leh, Lai Kuan Lee

**Affiliations:** Food Technology Program, School of Industrial Technology, Universiti Sains Malaysia, Gelugor 11800, Pulau Pinang, Malaysia; huiengleh@student.usm.my

**Keywords:** antioxidant, complementary medicine, lycopene, oxidative stress, type II diabetes mellitus

## Abstract

Nutrition is of utmost importance in chronic disease management and has often been described as the cornerstone of a variety of non-communicable diseases. In particular, type II diabetes mellitus (T2DM) represents a prevalent and global public health crisis. Lycopene, a bright red carotenoid hydrocarbon found in tomatoes and other red fruits and vegetables, has been extensively studied for its biological activities and treatment efficiency in diabetes care. Epidemiological investigations indicate that lycopene has potential antioxidant properties, is capable of scavenging reactive species, and alleviates oxidative stress in T2DM patients. This review aims to summarize the characteristics and mechanisms of action of lycopene as a potent antioxidant for T2DM. In addition, the evidence demonstrating the effects of lycopene on glycemic control and oxidative stress biomarkers in T2DM are also highlighted using animal and human studies as literature approach.

## 1. Introduction

Type II diabetes mellitus (T2DM), which accounts for 90% of diabetes cases, is a global public health crisis. The International Diabetes Federation (IDF) estimated that approximately 463 million adults worldwide are living with diabetes, and 4.2 million people died from diabetes in 2019 [1]. T2DM is a metabolic disease characterized by peripheral insulin resistance and impaired insulin secretion caused by dysfunction of the β-cell in the pancreas [2]. It is mostly seen in older adults, but it has increasingly affected children, adolescents, and younger adults as a consequence of rapid urbanization, unhealthy diets, and increasingly sedentary lifestyles. T2DM is often asymptomatic in the early stage and can remain undiagnosed for many years. Undiagnosed and poorly managed glucose levels are associated with life-threatening complications, such as cardiovascular disease (CVD), neuropathy, nephropathy, and retinopathy. Diabetes not only imposes a huge health burden but also has a substantial economic impact on countries and national health systems, due to the increased use of health services, loss of productivity, and the long-term support needed for the care and treatment of diabetic-related complications [3].

Numerous risk factors are known as contributors to the development of T2DM. Besides lifestyle and genetic factors, earlier epidemiological and animal studies have uncovered the impact of oxidative stress in the pathogenesis of T2DM and its complications [4,5,6,7]. In T2DM patients, hyperglycemia state favors free radical production through several pathways: activation of the polyol pathway, formation of advanced glycation end products (AGEs) and its receptors (RAGE), activation of the protein kinase C (PKC) pathway, and increased glucose influx through the hexosamine pathway. Overproduction of reactive species decreases antioxidant defense system and thus leads to the damage of redox equilibrium, subsequently increasing the risk of developing T2DM complications including heart disease, stroke, end-stage renal failure, blindness, and amputation [8].

T2DM is progressive and needs to be managed through medication and lifestyle modification, including a healthy diet and regular exercise [9]. The importance of antioxidant as an indispensable nutrient to protect against oxidative cell damage has been given much attention along with the increased incidence of diabetes worldwide. Given that synthetic antihyperglycemic agents and drugs have the potential to induce various side effects, numerous studies have suggested useful evidence of bioactive compounds from plant-based food in ameliorating diabetes and its complications. Lycopene, given its potent antioxidant properties, has received considerable interest among the researchers to study its role as a complementary antidiabetic agent [10]. Lycopene, a strong lipophilic carotenoid, is mostly present in tomatoes and tomato-based products. The antioxidative properties of lycopene have been attributed to its highly conjugated double bonds and a lesser influence by either the presence of cyclic or acyclic end groups in the structure. Much experimental evidence has provided the link between lycopene and diabetes-induced oxidative stress by measuring various biomarkers and lipid peroxidation products, including enzymatic endogenous antioxidants glutathione peroxidase (GPx), superoxide dismutase (SOD), and malondialdehyde (MDA) levels in the plasma or tissue samples [11,12,13].

Although experimental evidence has been reported to demonstrate the therapeutic effects of lycopene in diabetes [14], it should be noted that the underlying molecular mechanisms of action are far from being fully understood. Numerous factors may potentially affect its biological effects such as bioavailability, absorption, and metabolism functioning in vivo. Thus, this review summarizes (i) the characteristics and mechanism of action of lycopene as a potent antioxidant towards T2DM, (ii) biochemical pathways involving oxidative stress and T2DM, and (iii) the literature regarding the lycopene effects on glycemic control and oxidative stress biomarkers in T2DM.

## 2. Pathophysiology of T2DM

T2DM or non-insulin-dependent diabetes mellitus (NIDDM) is the most common form of diabetes. It is described as a complex endocrine and metabolic disorder in which the insulin receptors or other intermediates in the insulin signaling pathways within the body cells are insensitive to insulin. Eventually, glucose in the bloodstream fails to enter tissues thus leading to hyperglycemia or elevated blood glucose concentrations [5]. Insulin resistance as the key feature of T2DM, along with other clinical abnormalities such as hyperlipidemia, obesity, hypertension, and hyperinsulinemia contribute significantly to the development of T2DM. People suffering from T2DM are vulnerable to various short- and long-term complications, which often lead to their premature death. This multifactorial disease is a result of the interaction between genetic, environmental, and behavioral risk factors, which can be prevented and managed by adopting a healthy lifestyle, adhering to a medication regime, and maintaining good glycemic control.

The process of maintaining a blood glucose concentration at a steady-state level is called “glucose homeostasis” [15]. This is accomplished by the antagonistic effects of insulin and glucagon, through the regulation of insulin secretion, peripheral glucose uptake, and hepatic glucose production. The maintenance of a normal glucose concentration is achieved through the balance between the rate of glucose consumption and assimilation from the gut, the rate of glucose utilization by peripheral tissue (Krebs cycle, pentose phosphate pathway, the glycolytic pathway, glycogenesis), and endogenous production of glucose via gluconeogenesis and glycogenolysis [16]. Disruptions in glucose homeostasis will cause hunger, weakness, blurry vision, seizures, loss of consciousness, and coma in the situation of low blood concentration. Long-term elevated blood glucose concentrations will lead to hyperglycemia, a condition that leads to the progression of T2DM if left untreated.

T2DM is a multifactorial disease involving a combination of genetic and environmental factors such as obesity, physical inactivity, smoking, and alcohol consumption. The two main pathological defects in T2DM are impaired insulin secretion through a dysfunction of the pancreatic β-cells and impaired insulin action through insulin resistance [17]. Weight and obesity are the major factors causing the development of insulin resistance and T2DM progression. In obese individuals, excessive accumulation of triacylglycerol and fatty acid metabolites such as the long-chain acyl-CoAs, diacylglycerols and ceramides in the sarcoplasm of skeletal muscle causes a reduction in insulin signaling and glucose disposal rates [18].

## 3. Oxidative Stress in T2DM Pathogenesis

Hyperglycemia has been proposed as a cornerstone for the increased in vivo production of reactive oxygen species (ROS) and reactive nitrogen species (RNS). In the context of hyperglycemia, excessive ROS/RNS production, especially in the intra-mitochondrial environment, causes disturbances in cellular equilibrium and subsequent oxidative stress. Besides its potential in inflicting macromolecular damage, ROS and RNS also cause indirect damage in other cellular tissues by activating several cellular stress-sensitive pathways, including (i) activation of the polyol pathway, (ii) formation of advanced AGE and its receptors (RAGE), (iii) activation of the PKC pathway, and (iv) increased glucose influx through the hexosamine pathway [19,20,21]. This scenario has highlighted the role of hyperglycemia in reinforcing the relationship between oxidative stress and T2DM.

### 3.1. Glucose Influx through the Polyol Pathway

ROS production through the polyol pathway involved two processes:

(i) The reduction of glucose to sorbitol by aldose reductase (AR) with the help of its cofactor NADPH. In the state of hyperglycemia, about 30% of glucose is metabolized by the polyol pathway. The increased activity of AR in converting glucose has caused a depletion in its cofactor NADPH, which is also essential for the production of glutathione (GSH). GSH is an important cellular antioxidant that is capable of preventing ROS damage to cellular components. The decrease in GSH level has weakened the antioxidant capacity, thus favoring the condition for oxidative stress.
(ii) Resultant sorbitol is oxidized to fructose by the enzyme sorbitol dehydrogenase (SDH), with its cofactor NAD^+^. NAD^+^ is converted to NADH along with the oxidation process. The overproduction of NADH activates NADH oxidase (NOx) to produce superoxide anions [22,23].

### 3.2. Intracellular Production of AGEs

High fructose production in the polyol pathway accelerates AGEs formation [23]. Fructose, the end product of the polyol pathway, is converted to fructose-6-phosphate (F-6-P) by hexokinase. F-6-P is further converted to fructose-1,6-bisphosphate (F-1,6-P) by phosphate-fructokinase and forms dihydroxyacetone phosphate (DHAP). DHAP is interconvertible with glyceraldehyde-3-phosphate (GA3P) to generate methylglyoxal, leading to the formation of AGEs [24]. AGE is created through the nucleophilic addition reaction between the aldehyde or ketone moiety of glucose with the free amino groups of proteins. This reaction creates a Schiff base and eventually restructures into a skin collagen-linked fructosamine (Amadori product) and then to irreversible AGE [25]. AGEs, along with the generation of ROS, binds to RAGE to activate nuclear factor-kappa B (NF-κB) transcription factor [26], stimulate cell division, promote the release of proinflammatory cytokines such as IL-α and tumor necrosis factor-alpha (TNF-α), increase the expression of growth factor (TGFB), and subsequently lead to cellular and vascular dysfunction [27]. Thus, AGEs formation by increased glucose influx through the polyol pathway plays an important role in the pathogenesis of diabetic complications [28].

### 3.3. PKC Activation Pathway

The activation of PKC under hyperglycaemia condition mainly occurs through diacylglycerol (DAG)–PKC pathways. Cellular activities of PKC are upregulated by elevated DAG levels. De novo synthesis of DAG is derived from the glycolytic intermediate DHAP after reduction to glycerol-3-phosphate (G-3-P) and acylation to lysophosphatidic acid and phosphatidic acid (PA) [29]. Excessive activation of several PKC isoforms is associated with endothelial dysfunction, vasoconstriction, cell proliferation, angiogenesis, extracellular matrix expansion, activation of NF-κB, and mitogen-activated protein kinases (MAPKs), which may alter several transcription factors and gene expression. These PKC-induced changes in cellular functions and signal transduction pathways have been strongly implicated in the pathogenesis of diabetic complications [30,31].

### 3.4. Hexosamine Pathway

Under the hyperglycaemic condition, increased influx of glucose through hexosamine biosynthesis pathway (HBP) generates glucosamine 6-phosphate from F-6-P using glutamine: fructose-6-phosphate aminotransferase (GFAT), thus leading to the formation of the end product uridine diphosphate *N*-acetyl-glucosamine (UDP-GlcNAc). The elevated synthesization of UDP-GlcNAc drives intracellular *O*-glycosylation, along with *O*-GlcNAc transferase to generate glycoprotein, glycolipids, proteoglycans, and glycosylphosphatidylinositol anchors [32]. The dynamic and reversible post-translational protein modification usually on Ser/Thr residues are responsible in cellular signaling, alteration of transcription factors, cofactors in adipocytes, muscle cells, and pancreatic β-cells, which may alter gene expression leading to diabetic complications. For instance, HBP contributes to the pathogenesis of nephropathy by thickening the basement membrane, increased expression of plasminogen activator inhibitor (PAI)-1, and by upregulating transforming growth factor (TGF)-β, an autocrine/paracrine growth factor that causes the accumulation of extracellular mesangial matrix protein (ECM) (fibronectin, laminin, and collagen) [33].

### 3.5. Relationship between Oxidative Stress and T2DM

The relationship between T2DM and oxidative stress has been highlighted in numerous experimental studies through the measurement of oxidative stress biomarkers in T2DM patients. As shown in Table 1, the enzymatic antioxidant GPx was significantly decreased in T2DM patients compared to controls [34,35,36,37,38]. SOD activity was reduced in the study conducted by Al-Jiffri [34] and Mandal et al. [39], increased in the study by Aouacheri et al. [35], and remains unchanged in the study by George and colleagues [40]. On the contrary, pre-T2DM and T2DM patients showed higher lipid peroxidation levels as reflected by higher MDA and TBARS levels compared to the controls in majority of the studies. In terms of the effect of glycemic control on antioxidant capacity, reduced TAC and GPx along with an increased MDA level were observed in T2DM with good and poor glycemic control compared to controls [36]. T2DM patients with non-alcoholic steatohepatitis (NASH) had a significantly higher level of MDA compared to T2DM without NASH and healthy controls [41]. Another study investigating the antioxidant status among T2DM patients with and without cardiovascular complications (coronary heart disease, hypertension, and myocardial infarction) revealed a significantly decreased GPx and SOD in T2DM with cardiovascular complications compared to controls [42]. The study further revealed a weak positive correlation between enzymatic antioxidants (GPx and SOD) and glucose concentration among T2DM patients without cardiovascular complications.

## 4. Lycopene

Lycopene, also known as psi-carotene, belongs to the family of organic pigments known as carotenoid. Carotenoid is a lipid-soluble pigment synthesized by plants and microorganisms. Carotenoid comprises more than 700 compounds and is responsible for the yellow, orange, and red colors in many fruits and vegetables [43]. Approximately 90% of the carotenoids in the diet and human body are represented by β-carotene, α-carotene, lycopene, lutein, and cryptoxanthin [44].

### 4.1. Chemistry and Physical Properties

**Figure 1:**
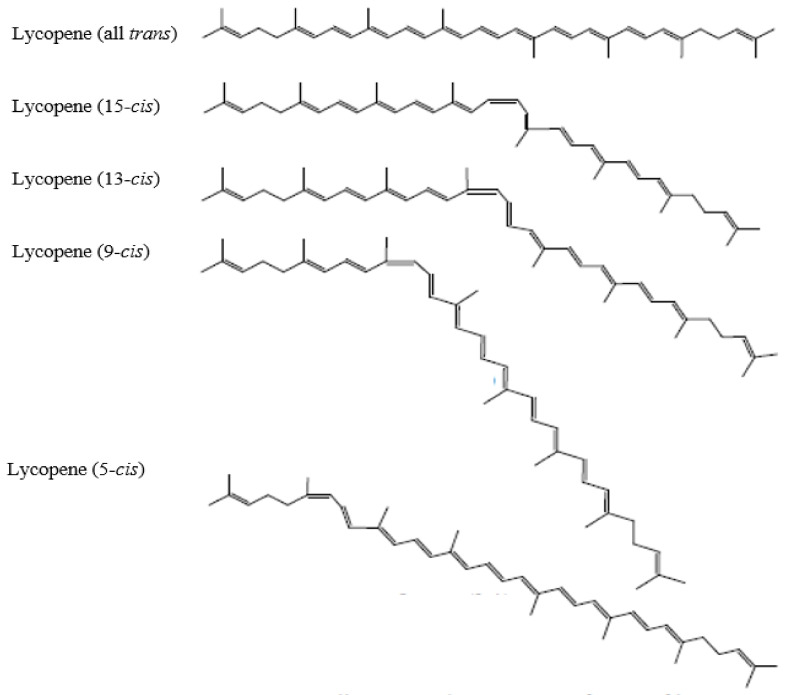
Chemical structure of lycopene [53].

Lycopene, with the molecular formula of C_40_H_56_, is a highly unsaturated open straight-chain hydrocarbon. It has a molecular weight of 536.85 g/mol and Chemical Abstract Service (CAS) Registry Number 502-65-8. Figure 1 shows the chemical structure of lycopene. Lycopene consists of 11 conjugated and 2 non-conjugated double bonds. The abundance of double bonds is critical for extensive isomerization, which can form up to 1056 theoretical *cis*-*trans* configurations [45,46]. Lycopene from natural plant sources is present primarily in the all-*trans* isomeric form, except for watermelon [47]. It undergoes mono- or poly-isomerization to *cis*-isomeric forms when interacting with light, thermal energy and chemical reactions [48]. Lycopene found in the human serum, breast milk, and tissues (liver, adrenal, adipose, prostate) mainly appears in *cis* isomeric form. Studies have shown that lycopene is stable under thermal processing and storage [49]. Among all the isomers, 5-*cis* lycopene was reported to be the most stable form, followed by all-*trans*, 9-*cis*, 13-*cis*, 15-*cis*, 7-*cis* and 11-*cis* [50]. Unlike some other carotenoids, lycopene lacks the terminal β-ionic ring in its structure and has no pro-vitamin A activity [51]. Hence, it appeared as the most potent antioxidant among other carotenoids. Among those, 5-*cis* isomer has the highest antioxidant properties, followed by 9-*cis*, 7-*cis*, 13-*cis*, 11-*cis*, and all-*trans* isomers [50]. In ripe tomato fruits, lycopene exists in an elongated, needle-like crystal form. It is a lipophilic compound with hydrophobic characteristics. Its acyclic structure and linear conjugated double bonds have made it more soluble in chloroform, hexane, benzene, and other organic solvents than in water [52]. The stability of lycopene is highly susceptible to light, oxygen, heat, acids, catalyst, and metal ions.

### 4.2. Dietary Sources

Lycopene is a naturally occurring pigment in tomatoes and to a lesser extent in some other foods such as pink grapefruit, red grapes, watermelon, red guava, apricots, red carrots, papayas, rosehip, wolfberry, and gac (*Momordica cochinchinensis*). It can also be obtained from certain algae and fungi. Tomatoes and tomato-based food products including sauce, juice, ketchup, and soup account for more than 85% of all dietary sources of lycopene for Americans [54]. Lycopene from processed tomato products which exposed to light, heat and in contact with oil showed higher bioavailability than unprocessed tomatoes. In detail, lycopene can be obtained from: (i) natural lycopene; (ii) synthetic lycopene; and (iii) lycopene from *Blakeslea trispora* [55].

#### 4.2.1. Natural Lycopene

Table 2 summarizes the average dietary exposure to lycopene from all sources. Lycopene can be found at high concentrations in many fruits and vegetables which are red in color, particularly tomatoes. Lycopene in tomatoes consists of 94–96% all-*trans*, 3–5% 5-*cis*, 0.1% 9-*cis*, 1% 13-*cis*, and <1% other *cis* isomers. Most lycopene in tomatoes can be found within the insoluble and fibrous parts, mainly tomato skin, which comprises 5 times more lycopene than the pulp [56]. Lycopene in tomato constitutes over 60% of the carotenoids, with other carotenoids found in tomatoes including δ-carotene (1–2%), γ-carotene (1–1.3%), neurosporene (7–9%), lutein (0.01–1.1%), phytoene (5.6–10%), phytofluene (2.5–3%), α-carotene (0.03%), and β-carotene (3–7%) [48]. Tomatoes also contain certain amounts of flavonoids (kaempferol, naringenin, quercetin, and hydrocinnamic acids), fibers (cellulose, pectins) and also a good source of vitamins and minerals including vitamin B6, C, E, biotin, folic acid, potassium and riboflavin.

#### 4.2.2. Synthetic Lycopene

Many processed foods are fortified with synthetic lycopene to increase the total dietary intake of this carotenoid. Synthetic lycopene is a highly purified product with 96% lycopene. Synthetic lycopene consists of all *trans*-lycopene (>70%) and approximately 3.5% other *cis* isomers. The manufacturing process usually involves the Wittig condensation of synthetic intermediates. Three different formulations of commercial lycopene preparations are lycopene 10%, lycopene 10 cold-water dispersible (CWD) in dark red powder form, and lycopene dispersion 20%. These synthetic lycopenes offer an alternative to the food industry to replace the extraction of lycopene from tomatoes, which have been widely used in breakfast cereals, bakery, convenience food, dairy, sauces, and sweets [45].

#### 4.2.3. Lycopene from *Blakeslea trispora*

The fungus *B. trispora* is involved in the biosynthesis of lycopene, and predominantly appeared in the all-*trans* isomeric form. The product, which is then formulated into a 2% or 5% sunflower oil suspension with α-tocopherol, forms lycopene oil suspension. The suspension is used as a food ingredient in fat spreads (2.0–5.0 mg/kg), milk and milk products (3.0–6.0 mg/kg), condiments, seasonings, relishes and pickles (all at 6.0 mg/kg), mustard (5.0 mg/kg), savory sauces and gravies (7.0 mg/kg), soups and soup mixes (6.0 mg/kg) and sugar, preservatives and confectionery (5.0 mg/kg), and dietary supplements [57]. Recently, the toxicological aspects of the chemicals employed for the production of lycopene from *B. trispora* is receiving aesthetic concern among the scientific communities, aligned with the increased demand of consumers for natural and safe food ingredients [58,59].

### 4.3. The Use of Lycopene and Acceptable Daily Intake (ADI)

Lycopene extracted from tomatoes has a strong deep red color. It is authorized for use as a food colorant in the USA (CDR 21 73.295), Australia, New Zealand (registered as 160d under Schedule 3 of Standard 1.3.1 in Australia New Zealand Food Standards Code), and the European Union (registered as E160d under EFSA; UK Food Standard Agency). In Japan, tomato color, defined as “a substance composed mainly of lycopene obtained from tomato fruits”, is permitted for use as a food additive under the Food Sanitation Law. Meanwhile, synthetic lycopene is currently not being approved as food coloring within the EU, but is considered as generally recognized as safe (GRAS) for use as a food ingredient by the FDA (GRAS notice No. GRN 000119). Non-synthetic lycopene (commonly referred to as “lycopene juice”) is used as colorant in food preparations, dairy products, non-alcoholic flavored drinks, cereal products, fish and meat products, and spreads to increase the visual appeal of food products. Synthetic lycopene is also added to some food and beverages as dietary supplement, infant formula, breakfast cereals, instant soup, low-fat dressing, nutrient bars and meal replacements, yogurt, meatless meat products, crackers, salty snacks, and drinks (i.e., juice drinks, dairy fruit drinks, and energy drinks) [59]. Safety evaluation of natural tomato oleoresin extract derived from food-processing tomatoes reported that the 50% lethal dose (LD_50_), derived from the acute oral toxicity study was greater than 5000 mg/kg body weight [60].

An acceptable daily intake (ADI) of 0.5 mg/kg BW/day using a safety factor of 100 for lycopene from all sources has been established by the EFSA panel on food additives, flavorings, processing aids and materials in contact with foods (AFC) in 2008 [57]. This guideline was concluded based on a one-year rat study, which established a no-observed adverse-effect level (NOAEL) of 50 mg/kg BW/day and a non-reversible increase in alanine transaminase (ALT).

## 5. Lycopene as Antioxidant

The antioxidant property of lycopene has been the main focus of research. The reactivity of lycopene with reactive species is related to its unique molecular and physical structure, including the highly conjugated double bonds which can be easily attacked by electrophilic reagents, and to a lesser extent influenced by either the presence of cyclic or acyclic end groups [61,62]. Among the carotenoids, lycopene is reported as the most efficient singlet oxygen (^1^O_2_) quencher [63] with the physical quenching rate constant (k_q_) of ^1^O_2_ = 3.1 × 10^10^ M^−1^ s^−1^. The quenching rate was reported as two times higher compared to β-carotene and 10 times higher compared to α-tocopherol. A comparison of the ^1^O_2_ quenching ability between lycopene and other carotenoids are described as: lycopene > y-carotene > astaxanthin > canthaxanthin > α-carotene > β-carotene > bixin > zeaxanthin > lutein > cryptoxanthin > crocin > α-tocopherol > lipoic acid > glutathione [64] (Table 3). The density functional theory study conducted by Zhang et al. [65] applied the optimization configurations of the ground and excited states of lycopene and oxygen, respectively. Another study provided evidence for the high capability of lycopene in preventing nitrogen dioxide-induced oxidation of lipid membranes and subsequent cell death compared to β-carotene [65].

### 5.1. The Mechanisms of Action of Lycopene in Scavenging Reactive Species

Lycopene has been reported to reduce lipid peroxidation by acting as a chain-breaking antioxidant [66]. This antioxidative role can be seen through its reaction with peroxyl radicals, a reactive species produced in the process of lipid peroxidation that can destruct lipophilic sections. Lycopene is forming new chain-carrying peroxyl radicals that are highly stable than ROS.

Tinkler and colleagues [66] conducted a physical chemistry technique based on singlet oxygen luminescence at about 1270 nm, and a biological cell membrane technique was used to study the quenching of singlet oxygen by lycopene bound to the surface of lymphoid cells. Interaction of lycopene with a peroxyl radical (ROO^•^) will result in adduct formation, the formation of resonance-stabilized carbon-centered radicals. This occurred when the peroxyl radical is attached to the polyene chain, the highly conjugated double bonds of lycopene forming a lycopene–peroxyl radical adduct (ROO–lycopene^•^) (Equation (1)) [66,67].

Lycopene + ROO^•^ → ROO–lycopene^•^ (1)

The compound ROO–lycopene^•^ acts as pro-oxidant by reacting with oxygen to form a new lycopene–peroxyl radical (ROO–lycopene-OO^•^) (Equation (2)). Subsequently, this intermediate (ROO–lycopene-OO^•^) can serve as an initiator for lipid peroxidation by reacting with lipid (RH) (Equation (3)) and forming another peroxyl radical (ROO^•^) with oxygen (Equation (4)), which is more highly stable than ROS. Nevertheless, the peroxyl radical–lycopene adduct (ROO–lycopene^•^) may also be terminated by reacting with other peroxyl radicals to form an inactive end product (Equation (5)).

ROO–lycopene^•^ + O_2_ → ROO–lycopene–OO^•^ (2)

ROO–lycopene–OO^•^ + RH → ROO–lycopene–OOH + R^•^ (3)

R^•^ + O_2_ → ROO^•^ (4)

ROO–lycopene^•^ + ROO^•^ → inactive products (5)

Lycopene’s chain structure with an extensive conjugated polyene system has increased its ability in scavenging ^1^O_2_ as shown in Equation (6) [68]:

^1^O_2_ + lycopene → ^3^O_2_ + ^3^lycopene (6)

^3^lycopene → lycopene + heat

It was also reported that lycopene reacts very rapidly with alkylthiyl radical (RS^•^) and glutathiyl radical (GS^•^) to generate lycopene-alkylthiyl radical adduct (RS-lycopene^•^) and lycopene-glutathiyl radical adduct (GS-lycopene^•^), at the absolute rate constant of 1.6 × 10^9^ M^−1^ s^−1^ and 4.8 × 10^8^ M^−1^ s^−1^, respectively (Equation (7)).

Lycopene + RS^•^ → RS–lycopene^•^ (7)

Lycopene + GS^•^ → GS–lycopene^•^

Lycopene in the process of scavenging radicals involves the electron transfer reactions as a result of the formation of lycopene cation radical (lycopene^+•^), anion radical (lycopene^−^^•^), or alkyl radical (lycopene^•^). For example, inactivation of nitrogen dioxide radical (NO_2_^•^) and trichloromethylperoxyl (CCl_3_O_2_^•^) converts lycopene into radical cations (Equation (8)), whereas the interaction of lycopene with superoxide radical (O_2_^●−^) forms lycopene anion radical [69] (Equation (9)).

NO_2_^•^ + Lycopene → NO_2_^−^ + Lycopene^+•^ (8)

CCl_3_O_2_^•^ + Lycopene → [CCl_3_O_2_^−^ Lycopene]^•^ → CCl_3_O_2_^−^ + Lycopene^+•^

Lycopene + O_2_^•−^ → lycopene^•−^ + O_2_ (9)

Moreover, the formation of both lycopene adducts radical and lycopene cation radical is generated through the interaction between lycopene and the thiylsulfonyl radical (RSO_2_^•^), at an absolute rate constant of 1.26 × 10^9^ M^−1^ s^−1^, as shown in Equation (10):

RSO_2_^•^ + lycopene → [RSO_2_^−^ lycopene]^•^ → RSO_2_^−^ + lycopene^•+^ (10)

In contrast, lycopene also acts as a hydrogen donor to reduce the radical. This reaction is known as hydrogen abstraction, as shown in Equation (11) [70]:

Lycopene + ROO^•^ → Lycopene^•^ + ROOH (11)

### 5.2. Synergistic Effect of Lycopene with Other Antioxidants

The reactivity of lycopene with ROS depends not only on their molecular and physical structure, but also on their location or site of action within the cells, concentration and the partial pressure of oxygen, as well as their ability to interact with others [71]. Lycopene is a highly lipophilic carotenoid located within the hydrophobic core of lipoprotein, therefore exerting higher capability in scavenging free radicals in a hydrophobic environment. However, as a lipid-soluble radical scavenger, lycopene has less interaction with aqueous phase radicals. It was suggested that the scavenging activity of lycopene in the lipoprotein particle can be maximized by its interaction with other carotenoids, for instance, α-tocopherol located near the membrane surface. Specifically, α-tocopherol scavenges lycopene-derived peroxyl radicals (ROO–lycopene–OO^•^) via hydrogen atom donation, giving rise to a relatively stable α-tocopherol radicals (TO^•^) (Equation (12)) [72]. Additionally, lycopene helps in repairing α-tocopherol radicals as shown in Equation (13) [73].

α-TOH + ROO–lycopene–OO^•^ → ROO–lycopene–OOH + TO^•^ (12)

Lycopene + TOH^+^^•^→ TOH + Lycopene^+^^•^ (13)

On the other hand, α-tocopherol might play a role in regenerating intact lycopene by reducing lycopene cation radical (TOH^+•^) (Equation (14)) [74].

α-TOH + Lycopene^+^^•^ → α-TO^•^ + Lycopene (14)

In this context, the synergistic effects between lycopene and α-tocopherol in different cellular locations have provided a greater resistance for lipid and lipoproteins against oxidative damage [75]. Synergistic interactions among lycopene and other carotenoids have also been demonstrated in multiple studies. For example, a study using multilamellar liposomes reviewed an inhibitory effect of lycopene and lutein towards diene hydroperoxides produced from linoleic methyl ester with 2,2′-azobis (2,4-dimethylvaleronitrile) (AMVN)-induced oxidation [76], whereas the interaction of lycopene and vitamin C, E, and β-carotene showed a high scavenging activity on 2,2-diphenyl-1-picrylhydrazyl (DPPH) radical than their individual antioxidant activity [77].

## 6. Lycopene Consumption and T2DM

### 6.1. Lycopene Status in T2DM Patients

The lycopene status of T2DM patients from different populations has been studied extensively by previous researchers. In a cross-sectional surveillance study, the lycopene level of 24,377 Korean adults (19–74 years) was assessed using 24-h dietary recall. The result showed the dietary lycopene intake was significantly higher in non-T2DM men compared to T2DM men [78,79]. In a case-control study, lycopene intake in T2DM patients was significantly lower compared to age-matched healthy controls. The study further explicated that subjects with proliferative diabetic retinopathy had significantly lower lycopene levels than subjects without diabetic retinopathy or with non-proliferative diabetes [80,81]. This result is in accordance with a community-based cross-sectional study in Australia, which demonstrated a significantly lower level of lycopene in the T2DM-retinopathy group [82,83]. Moreover, Ford et al. [84] reported that the United States (US) population with newly diagnosed T2DM had a significantly lower level of lycopene compared to the US adults with scarce glycemic control. Another study investigating the lycopene status among T2DM patients in Germany revealed the plasma concentration of lycopene was significantly lower in very old T2DM patients (mean age 75.7 ± 0.8 years) as compared to healthy controls. Also, a significant inverse correlation between age and the level of lycopene was reported in the study [85].

### 6.2. Animal Studies: Lycopene Effects on Glycemic Control and Oxidative Stress Biomarkers

The antidiabetic effect of lycopene has been studied in different animal models with various outcomes (Table 4). In diabetic rat models (streptozotocin (STZ)-induced), oral administration of lycopene significantly decreased blood glucose levels [6,80,86,87,88,89,90,91,92,93,94,95,96,97,98], reduced HbA1c levels [6,7,92], and increased insulin concentrations [86,89,90,95,96].

Besides the glucose-lowering and insulin-elevating effects, animal studies also demonstrated that lycopene prevents oxidative damage in diabetic rat models (Table 5). The antioxidant effect mainly occurs by enhancing the activities of antioxidant enzymes and increasing the level of non-enzymatic antioxidants. Indeed, the mechanism of action of lycopene is probably not only attributed to its scavenging mechanism, but rather due to the molecule itself to induce enzymatic defenses. Overall, such effect was accompanied by a decrease in the formation of ROS (H_2_O_2_) [78], reductions in MDA concentrations [6,91,95,96,99,100,101,102], and elevation of enzymatic antioxidants [78,82,87,91,92,93,94,95,96,97,98,100,101].

### 6.3. Human Studies: Lycopene Effects on Glycemic Control

Table 6 summarizes the human studies investigating the effects of lycopene on glycemic control in T2DM. In 2010, Li et al. [81] demonstrated that HbA1c was negatively correlated with lycopene. Coyne et al. [102] reported a significant reduction in plasma glucose and fasting insulin concentrations with increased serum lycopene in T2DM patients. However, She et al. [103] did not find a significant association between HbA1c and lycopene level in a sample of 40 T2DM participants. Bose and Agrawal [104] observed no significant changes in FBG and HbA1c levels for T2DM patients following a 30-day supplementation of ripe cooked tomatoes (200 g tomatoes/day). Similarly, Upritchard et al. [4] supplemented T2DM patients with 500 mL of tomato juice along with Vitamin E and C for 4 weeks, and reported that lycopene supplementation did not affect plasma glucose concentration. Very recently, HbA1c and FPG levels were found to decrease significantly with the higher lycopene intake [105]. The combined application of cross-sectional, case-control, prospective cohort, and randomized placebo-controlled trials generated a discrepancy in outcomes. This disagreement has been attributed to the wide selection of food sources to represent the lycopene intake in the model, disease state, and the sample size of the study.

### 6.4. Human Studies: Lycopene Effects on Oxidative Stress Biomarkers and Risk of T2DM

Lycopene-based dietary therapy indicated a significant role in the reduction of oxidative damage and improvement of LDL oxidation. The health benefits of lycopene on oxidative damage in human studies are depicted in Table 7. Accordingly, Singh [14] conducted a 3-month-long study to investigate the effect of lycopene administration (4 mg once daily) in T2DM subjects. The levels of MDA, SOD, GPx, GSH, glutathione reductase (GR), and xanthine dehydrogenase (XOD) were determined in blood samples to evaluate the oxidant–antioxidant status. The study revealed significant elevations in the SOD, GSH, GPx, and GR, and a further decrease of MDA and XOD levels in the lycopene-ingesting T2DM patients in comparison to T2DM patients who did not receiving lycopene. Likewise, long-term supplementation of 200 g cooked tomatoes per day in T2DM patients showed significant improvement in the levels of antioxidant enzymes (SOD, GSH, GPx, and GR) and decreased lipid peroxidation rate (MDA level) after 30 days of tomato supplementation [104]. Neyestani et al. [5] demonstrated that administration of 10mg/day of lycopene for 8 weeks significantly increased the serum lycopene levels in T2DM patients, further preventing oxidative damage by inhibiting MDA-LDL formation and increasing TAC level. In addition, to investigate the synergic effects of lycopene and other antioxidants on oxidative stress, 57 T2DM patients were randomized to receive tomato juice (500 mL/day) supplementation along with vitamin E (800 U/day) and vitamin C (500 mg/day), or placebo treatment for 4 weeks [104]. The finding indicated that short-term supplementation of commercial tomato juice increased plasma lycopene levels nearly three folds, and the intrinsic resistance of LDL to oxidation by 42% in well-controlled T2DM, which were almost as effective as supplementation with a high dose of vitamin E. Conclusively, supplementation of lycopene in the short and long term attenuates oxidative damage by increasing the antioxidant enzyme level and reducing lipid peroxidation rate in the individual with T2DM.

On the contrary, some studies reported null effects of lycopene on T2DM. For example, a prospective study in Korea failed to show a correlation between dietary lycopene and the incidence of T2DM, even though lycopene intake was significantly higher in non-diabetic subjects than in diabetic patients [79]. In a European Prospective Investigation into Cancer and Nutrition-Netherlands (EPIC-NL) cohort study (*n* = 37,846), Sluijs et al. [106] demonstrated that lycopene intake was not associated with a reduced risk of T2DM. A similar result was depicted in a nested case-control study [107]. After 10 years of follow-up, the study showed no prospective association between baseline plasma lycopene, as assessed by using FFQ, with the risk of T2DM in middle-aged and older women from the United States. Another prospective study demonstrated that dietary lycopene did not reduce the risk of T2DM in a Finnish cohort of men and women [11]. In Asia, a cross-sectional study of the Chinese urban population also reported that lycopene has no protective role on T2DM [103].

## 7. Mechanisms of Action of Lycopene in T2DM

Lycopene could diminish oxidative damage by scavenging oxidized species and enhancing the antioxidative enzyme activity in T2DM, as evidenced in the animal experiments, and observational and epidemiological studies. It has been proposed that overproduction of ROS could downregulate the antioxidant defense mechanisms, leading to oxidative imbalance. Accordingly, lycopene treatment could upregulate the expression of CAT, SOD, and GPx, and reduce the levels of MDA in the pancreatic tissues [101], in the diabetic kidney [80], and in the furan-induced ovarian tissue injury [97]. Another study found that lycopene attenuates oxidative stress by decreasing serum Ox-LDL and liver thiobarbituric acid reactive substances (TBARS), and increased the levels of CAT and non-protein sulfhydryl groups in the liver of diabetic rats [82]. Additionally, the interaction between AGEs and its receptor, RAGEs, has been implicated in the oxidative stress-induced phosphatidylinositol-3-kinase (PI3K)/protein kinase B (AKT) (PI3K/Akt) signaling activation. Treatment of lycopene (20 mg/kg/day) for 8 weeks has been shown to promote Akt phosphorylation in diabetic renal tissue [80]. Similarly, 10mg/kg/d of lycopene supplementation for 5 weeks decelerated the ribose-induced AGE formation in HK2 cells and rat kidneys, thereby downregulating the expression of RAGE and protecting against diabetic nephropathy [11].

Moreover, it has been shown that vascular endothelial dysfunction and the number of endothelial progenitor cells (EPCs) are important risk factors for the development of vascular complications in T2DM. Zeng et al. [108] reported that lower cell proliferation, migration, adhesion, and in vitro vasculogenesis capacity, as well as increased EPC’s apoptosis, were observed in the high glucose rats group. Lycopene treatment inhibits high glucose-induced EPC injury by inhibiting ROS generation and downregulating phosphorylation of p38 mitogen-activated protein kinases (p38 MAPK). Lycopene also protects EPCs from apoptosis and oxidative autophagy induced by AGEs, as demonstrated in the T2DM rats [109]. Furthermore, lycopene supplementation (4 mg/kg) for 3 months prevents diabetic retinopathy by decreasing TNF-κB and TNK-α level and increasing total glutathione levels (TGSH) and total antioxidant status (TAS) [110]. Also, supplementation of lycopene-rich tomato extract at a concentration of 0.2, 0.4, and 0.8% may dose-dependently inhibit cataractogenesis by reducing aldose reductase activity and upregulating lens protein and GSH levels [98].

Guo et al. [99] suggested that lycopene upregulated heme oxygenase-1 (HO-1) mRNA levels in the diabetic kidneys, thereby maintaining kidney metabolic homeostasis. Notably, HO is a vital enzyme in heme catabolism that mediates the anti-oxidative and anti-inflammatory characteristics through modulating the interleukin 10 receptor 1 (IL-10/1R) pathway. The antioxidative effect of lycopene is also evident in the reduction of 8-hydroxy-2′-deoxyguanosine (8-OHdG) levels. The reduction was typical after the treatment with low, medium, and high doses (10, 20, 40 μM) of lycopene under high glucose conditions, demonstrating that lycopene scavenged free radicals, indirectly alleviating oxidative stress [111].

It is worthwhile to mention that lycopene is able to not only increase the peripheral antioxidative capacity, but also preserve glycemic control and protect against obesity in T2DM. Long-term hyperglycemic and insulin resistance could lead to glucose utilization disorders, which in turn causes excessive accumulation of FFAs and lipids in the bloodstream [112]. Lycopene intervention was demonstrated to regulate the metabolism of glycolipid in diabetic rats by decreasing FBG, glycosylated hemoglobin (GHb), and glycated low-density lipoprotein (Gly-LDL) levels. Lycopene has been proven to improve glucose metabolism by reducing Ox-LDL, thus reducing the occurrence of autonomic oxidation of glucose and lipid peroxidation reaction [97]. Li et al. [80] reported that lycopene acts as a lipid-lowering agent that significantly decreases total cholesterol (TC), triglyceride (TG), and low-density lipoprotein cholesterol (LDL-C), while at the same time increasing high-density lipoprotein cholesterol (HDL-C) in diabetic renal tissues. Lastly, lycopene treatment has been reported to reduce vacuolization of the islets of Langerhans and the loss of insulin-secreting cells leading to reduced blood glucose levels in diabetic rats [90].

## 8. Conclusions

The gradual increase of T2DM incidence has resulted in the elucidation of several dietary approaches for proper diabetes management. From evidence in the literature, it can be inferred that the antioxidant properties of lycopene contributes to the prevention and complementary therapy for T2DM through a synergistic coupling effects of decreasing oxidative stress biomarkers, as well as inducing antioxidant defense mechanisms. Lycopene consumption beneficially contributes to protect against T2DM in animal studies. However, epidemiological observations and large-scale population studies using human models have revealed a mixed association between lycopene intake and T2DM. Therefore, properly designed clinical studies are warranted to clarify and validate the potential of lycopene in ameliorating diabetic conditions.

## Figures and Tables

**Table 1:** Clinical studies on peripheral antioxidant capacity in pre-T2DM, T2DM with and without complications.

No.	Study Population	Sample	Biomarkers	Observations	Reference
1	T2DM with CVD (*n* = 69) T2DM without CVD (*n* = 48) Control (*n* = 42)	erythrocyte	GPx SOD	↓ Decreased GPx and SOD in T2DM with CVD	[42]
2	T2DM (*n* = 20) Control (*n* = 20)	plasma and erythrocyte	GPx	↓ Decreased erythrocyte-GPx and plasma-GPx in T2DM	[38]
3	T2DM (*n* = 57) Control (*n* = 41)	serum	SOD TBARS	↑ Increased TBARS in T2DM No differences in SOD between T2DM and control	[40]
4	T2DM (*n* = 59) Control (*n* = 48)	serum	GPx SOD MDA	↓ Decreased GPx in T2DM ↑ Increased SOD in T2DM ↑ Increased MDA in T2DM	[35]
5	T2DM and NASH (*n* = 60) T2DM without NASH (*n* = 55) Control (*n* = 50)	serum	MDA	↑ Higher MDA in T2DM and NASH	[41]
6	T2DM (*n* = 115) Control (*n* = 32)	plasma	GPx	↓ Lower GPx level in T2DM	[37]
7	T2DM (*n* = 100) Control (*n* = 100)	serum	GPx SOD MDA	↓ Decreased GPx and SOD level in T2DM ↑ Increased MDA in T2DM	[34]
8	T2DM (*n* = 83) Control (*n* = 81)	serum	SOD MDA	↓ Lower SOD level in T2DM ↑ Higher MDA level in T2DM	[39]
9	Controlled-T2DM (*n* = 80) Uncontrolled T2DM (*n* = 80) Control (*n* = 100)	serum	GPx MDA	↓ Reduced GPx in controlled and uncontrolled T2DM; ↑ Increased MDA in controlled and uncontrolled T2DM	[36]

CVD: cardiovascular disease; GPx: glutathione peroxidase; MDA: malondialdehyde; NASH: non-alcoholic steatohepatitis; SOD: superoxide dismutase; T2DM: type II diabetes mellitus.

**Table 2:** Average dietary exposure to lycopene from all sources.

Source of Lycopene	Average (mg/day)	High (mg/day)	Reference
Naturally occurring	0.5–5	8–20	[57]
Fortified foods	8–19	23–37	[45]
Supplements	0 (no supplement use)	8–15	[45]
Food color	2–6	11–23	[57]

AFC: Opinion of the scientific panel on food additives, flavorings, processing aids and materials in contact with food; EFSA: European Food Safety Authority based on the 97.5th percentile intake estimates.

**Table 3:** The singlet oxygen (^1^O_2_) quenching ability of lycopene, other carotenoids and major antioxidants.

Compound ^a^	Number of Conjugated Carbon-Carbon Double Bonds ^b^	Terminal Rings	Quenching Rate Constant, (k_q_ L mol^−1^ s^−1^)	Relative Rates
Lycopene	11	0	3.1 × 10^10^	103
γ-Carotene	11	1	2.5 × 10^10^	83
Astaxanthin	11(2)	2	2.4 × 10^10^	80
Canthaxanthin	11(2)	2	2.1 × 10^10^	70
α-Carotene	10	2	1.9 × 10^10^	63
Bixin	9(2)	0	1.4 × 10^10^	47
β-Carotene	11	2	1.4 × 10^10^	47
Zeaxanthin	11	2	1.0 × 10^10^	33
Lutein	10	2	0.8 × 10^10^	27
Cryptoxanthin	11	2	0.6 × 10^10^	20
Crocin	7(2)	0	0.11 × 10^10^	3.7
α-Tocopherol	n.c.^c^	n.c.	0.03 × 10^10^	1
Lipoic acid	n.c.	n.c.	0.0138 × 10^10^	0.46
Glutathione	n.c.	n.c.	0.00024 × 10^10^	0.008

^a^ Names of carotenoids are given in italics. ^b^ Number of conjugated double bonds listed within parentheses. ^c^ n.c.: not compared for non-carotenoid compounds.

**Table 4:** Animal studies on the effects of lycopene on glycemic control in T2DM.

No.	Population	Treatment/Method	Effects	Reference
1	STZ-induced diabetic Wistar-Albino male rats	Oral administration of 10 mg/kg/day lycopene in corn oil for 28 days	Reduction of blood glucose level and HbA1c %	[6]
2	STZ-induced diabetic Wistar-Albino Rats	Administration of 10/mg/kg/day of lycopene for 28 days	Reduction in HbA1c %	[7]
3	STZ-induced diabetic mice	Lycopene supplementation (40, 80 mg)	Decreased serum blood glucose concentration	[80]
4	STZ-induced Albino Wistar rats	Supplementation of ripe and unripe tomato (10%) for 14 days	Increased insulin level (37%) Decreased glucose concentration (33%)	[86]
5	STZ-induced diabetic rats	Single dose of 90 mg/kg/bw tomato-extract lycopene	Decreased FBG levels	[87]
6	Wistar Rats	Lycopene niosomes (100 and 200 mg/kg/bw for 14 days)	Decreased FBG levels	[88]
7	STZ-induced diabetic male Balb/c mice	Oral administration of lycopene dose (100, 200 mg/kg/bw) for 10 days	Decreased in FBG levels; No changes in serum insulin levels	[89]
8	STZ-induced diabetic rats	Lycopene + caffeine administration by oral gavages for 1 month	Decreased blood and urine FBG levels; Increased serum insulin levels	[90]
9	STZ-induced diabetic rats	Administration of lycopene (10, 30, 60 mg/kg/bw) for 30 days	Decreased FBG levels	[91]
10	STZ-induced diabetic rats	Administration of 4 mg/kg/bw of lycopene	No significant changes in HbA1c levels; Decreased in FBG levels	[92]
11	STZ-induced diabetic rats	Oral administration of 10 mg/kg/bw lycopene for 3 weeks	Reduction of blood glucose levels by 25%	[93]
12	STZ-induced male Sprague-Dawley rats	Administration of lycopene (10, 30, 60 mg/kg/d) for 8 weeks	Decreased FBG levels	[94]
13	STZ-induced diabetic rats	Administration of lycopene at the dose of 2.5 mg/kg/bw for 7 days	Reduction in serum glucose levels; Increased serum insulin levels	[95]
14	STZ-induced diabetic Wistar rats	Oral administration of lycopene in sunflower oil at a dose of 4mg/kg/bw for 8 weeks	Decreased FBG levels; Increased plasma insulin concentration	[96]
15	STZ-induced diabetic Wistar Rats	Oral administration of lycopene (10, 20 and 40 mg/kg/bw) for 4 weeks	Decreased FBG levels	[97]
16	STZ-induced diabetic rats	Oral administration of lycopene oil solution (10 mg/kg or 20 mg/kg/bw) for 10 weeks	Decreased FBG levels	[98]

FBG: fasting blood glucose; HbA1c: glycated hemoglobin; STZ: streptozotocin.

**Table 5:** Animal studies on the effects of lycopene on antioxidant and oxidative stress biomarkers in T2DM.

No.	Population	Treatment	Effects on Oxidative Stress Biomarkers	Reference
1	24 STZ-induced diabetic Wistar rats	Oral administration of lycopene in sunflower oil at a dose of 4 mg/kg/bw for 8 weeks	Increased SOD, CAT and GPx activities in erythrocytes Decreased GSH and NO levels (plasma) and GSH levels (brain tissue); Decreased in brain tissue MDA levels but no significant effect in plasma MDA levels	[6]
2	40 STZ-induced diabetic male Balb/c mice	Oral administration of lycopene (100, 200 mg/kg/bwt) for 10 days	Decreased in ROS levels in serum, liver and pancreas tissues; Decreased in SOD, CAT, and GPx; Prevent increase in LPO level (liver, pancreas)	[7]
3	STZ-induced rats	Single dose of 90 mg/kg/bw of tomato-extract lycopene	Decreased H_2_O_2_ formation; Increased CAT, SOD and GPx	[78]
4	STZ-induced diabetic Wistar Rats	Oral administration of 90 mg/kg curcumin + 45 mg/kg lycopene in yogurt	Increased CAT levels; Decreased serum Ox-LDL and liver TBARS	[82]
5	STZ-induced diabetic rats	Single dose of 90 mg/kg/bw tomato-extract lycopene	Increased CAT, SOD and GPx	[87]
6	STZ-induced diabetic rats	Administration of lycopene (10, 30, 60 mg/kg) for 30 days	Increased aortic SOD activity; Decreased MDA levels	[91]
7	STZ-induced male diabetic rats	Administration of 4 mg kg^−1^ bw lycopene for 28 days	Increased GPx, SOD, CAT and GST levels in liver tissue Decreased MDA level in liver tissue	[92]
8	STZ-induced diabetic rats	Oral administration of 10mg/kg/bw lycopene for 3 weeks	Reduction of LPO rate and NO in the plasma	[93]
9	STZ-induced male Sprague–Dawley rats	Administration of lycopene (10, 30, 60 mg/kg/d) for 8 weeks	Decreased MDA levels and increased SOD activities	[94]
10	60 STZ-induced diabetic male Sprague–Dawley rats	Administration of 20 mg/kg/day lycopene by oral gavage tube for 8 weeks	Increased SOD activity; Decreased kidney MDA levels	[95]
11	STZ-induced diabetic nephropathy mice	Lycopene supplementation (40, 80 mg)	Augmented bioactivities of SOD, GPx; Reduction of MDA level	[96]
12	STZ-induced diabetic Wistar Rats	Oral administration of lycopene oil solution (10 mg/kg or 20 mg/kg/bw) for 10 weeks	Increased GPx and SOD Decreased MDA level in pancreas	[97]
13	Diabetic sand rats	Administration of natural tomato extract at 0.2% in the diet for 5 weeks	Increased GSH levels	[98]
14	STZ-induced diabetic Wistar Rats	Oral administration of graded dose of lycopene (10, 20 and 40 mg/kg bw) for 4 weeks	Decreased erythrocyte MDA concentration	[99]
15	STZ-induce female Wistar-Albino diabetic rats	Administration of 4 mg/kg/bw lycopene for 28 days	Increased CAT, SOD, GPx and GST enzymes activities; Decreased MDA level	[100]
16	STZ-induced diabetic Wistar Rats	Oral administration of lycopene dose (0, 5, 10 and 15 mg/kg/bw) for 10 weeks	Increased CAT, SOD and GPx Decreased MDA level in pancreas	[101]

CAT: catalase; GPx: glutathione peroxidase; GSH: glutathione; GST: glutathione-S-transferase; LPO: lipid peroxidation; MDA: malondialdehyde; NO: nitric oxide; Ox-LDL: oxidized low-density-lipoprotein; ROS: reactive oxygen species; SOD: superoxide dismutase; STZ: streptozotocin; TBARS: thiobarbituric acid reactive substances.

**Table 6:** Effect of lycopene on glycemic control in T2DM (Human studies).

No.	Study Design	Subjects Characteristics	Intervention	Effects on T2DM	References
1	Randomized placebo-controlled trial	T2DM (*n* = 57) Mean age 63 ± 8 years	Tomato juice (500 mL/day) + vitamin E (800 U/day) and vitamin C (500 mg/day) for 4 weeks	No changes in plasma glucose concentration	[4]
2	Case-control study	T2DM (*n* = 71) Non-T2DM (*n* = 23) Age > 50 years	Dietary Intake	Negative correlation between HbA1c and serum lycopene	[81]
3	Prospective cohort study	Total, *n* = 1597 T2DM (*n* = 132) Age ≥ 25 years	Dietary Intake	Reduced plasma glucose and fasting insulin concentrations with increased serum lycopene	[102]
4	Cross-sectional study	T2DM (*n* = 190) T2DM + DR (*n* = 272) Control (*n* = 285)	Dietary Intake	No significant association between HbA1c and lycopene	[103]
5	Case-control study	T2DM (*n* = 40) Control (*n* = 50) Age 35–55 years	Ripe tomatoes (cooked) (200 g/day) for 30 days	No significant changes in fasting blood sugar and HbA1c levels	[104]
6	Case-control study	T2DM (*n* = 87) Control (*n* = 122)	Dietary intake	HbA1c and FBG levels decreased significantly with higher lycopene intake	[105]

DR: diabetic retinopathy; HbA1c: glycated hemoglobin; T2DM: type II diabetes mellitus.

**Table 7:** Human studies on the effect of lycopene on oxidative stress and risk of T2DM.

No	Subjects Characteristics	Intervention	Effect after Lycopene Supplementation	References
1	T2DM (*n* = 57) >75 years	Tomato juice (500 mL/day) + vitamin E (800 U/day) and vitamin C (500 mg/day) for 4 weeks	↓ LDL oxidation	[4]
2	T2DM (*n* = 35) 54 ± 9 years	Lycopene supplementation (10 mg/d) or placebo for 8 weeks	↑ TAC levels; Inhibit MDA-LDL formation	[5]
3	T2DM (*n* = 4304) 40–69 years	Dietary intake	No association between lycopene and risk of T2DM	[11]
4	Total (*n* = 35,784) ≥45 years	131-item-validated semi-quantitative FFQ	No association between either dietary lycopene or lycopene-containing foods and the risk of T2DM	[13]
5	T2DM (*n* = 50) T2DM + Lycopene (*n* = 50) Control (*n* = 50) 48 ± 6 years	Oral administration of lycopene (4 mg once daily for 3 months)	↑ SOD, GPx, GR and GSH levels in T2DM + lycopene ↓ MDA and XOD in T2DM + lycopene	[14]
6	T2DM (*n* = 603) Control (*n* = 23,774) 19–74 years	24-h dietary recall	No association between lycopene intake and reduced risk of T2DM	[79]
7	T2DM (*n* = 40) Control (*n* = 50) 35–55 years	Supplementation with cooked tomato, 200 g for 30 days	↑ SOD, GPx, GR, GSH ↓ MDA	[104]
8	Total (*n* = 37,846) 49.1 years	Validated FFQ	No association between lycopene intake and reduced risk of T2DM	[106]

FFQ: food frequency questionnaire; LDL: low density lipoprotein; MDA; malondialdehyde; GPx: glutathione peroxidase; GR: glutathione reductase; GSH: glutathione; SOD: superoxide dismutase; TAC: total antioxidant capacity; T2DM: type II diabetes mellitus; XOD: xanthine dehydrogenase.

## Data Availability

Not applicable.
